# Metastatic rectal adenocarcinoma in the mandibular gingiva: a case report

**DOI:** 10.1186/s12957-016-0958-6

**Published:** 2016-07-29

**Authors:** Masato Watanabe, Masanori Tada, Takafumi Satomi, Daichi Chikazu, Masashi Mizumoto, Hideyuki Sakurai

**Affiliations:** 1 Department of Oral and Maxillofacial Surgery, Tokyo Medical University Ibaraki Medical Center, 3-20-1 Chuo, Ami-machi, Inashiki-gun, Ibaraki 300-0395 Japan; 2 Department of Oral and Maxillofacial Surgery, Tokyo Medical University Hospital, 6-7-1 Nishishinjuku, Shinjuku, Tokyo, 160-0023 Japan; 3 Department of Radiation Oncology, Faculty of Medicine, University of Tsukuba, 2-1-1 Amakubo, Tsukuba, Ibaraki 305-8576 Japan

**Keywords:** Metastasis, Gingiva, Rectal adenocarcinoma

## Abstract

**Background:**

Oral metastatic tumor from a rectal adenocarcinoma is very uncommon. The primary site is usually assumed based on the past clinical history. In the case of oral metastatic tumors, they commonly have a poor prognosis because often they have already spread to other sites.

**Case presentation:**

We present the case of a 64-year-old male patient with secondary metastasis to the mandibular gingiva via lung metastasis after the surgical resection of a primary rectal adenocarcinoma. The gingival lesion grossly appeared as a swollen mass, making mastication difficult. The patient received palliative radiotherapy for the mandibular mass lesion. However, tumor reduction was accompanied by the development of pneumonia and deterioration of the patient’s cachexia. Thus, the radiotherapy was discontinued but the patient died 2 months postradiotherapy. In the long term after its primary resection, the rectal adenocarcinoma was deduced to have finally metastasized to the oral region.

**Conclusions:**

In this case, we consider a distant secondary metastasis to the oral region from a rectal malignancy. In such cases, careful clinical and pathologic evaluations are necessary, with careful consideration of the inclusion of palliative treatment in the therapeutic management.

## Background

Metastatic tumors to the oral cavity are rare and account for approximately 1 % of all oral malignant neoplasms [1]. The primary site is usually assumed based on the past clinical history. Most patients were previously diagnosed in relation to a primary neoplasm and were treated accordingly. However, 23 % of oral metastatic tumors represent the first clinical sign of the metastatic process [2]. The most common primary sites are the lungs in men and the breast in women [2]. The peak incidence of oral metastasis is observed during the age of 50–70 years [2]. In the oral cavity, hard tissues such as the jawbone are more commonly affected than soft tissues such as the gingiva [3]. The gingivae (55 %) followed by the tongue (30 %) are the most common soft tissue sites affected by metastatic tumor [4]. Colonic carcinoma is one of the most common malignant neoplasms worldwide. Its usual sites of metastasis are the regional lymph nodes, liver, lungs, cutis, vagina, myocardium, breast, and prostate. Metastasis to the gingiva is particularly rare. Here, we report the case of a metastatic rectal adenocarcinoma spreading to the mandibular gingiva via lung metastasis over the long clinical term of a patient following surgical tumor resection at the primary site.

## Case presentation

**Fig. 1:**
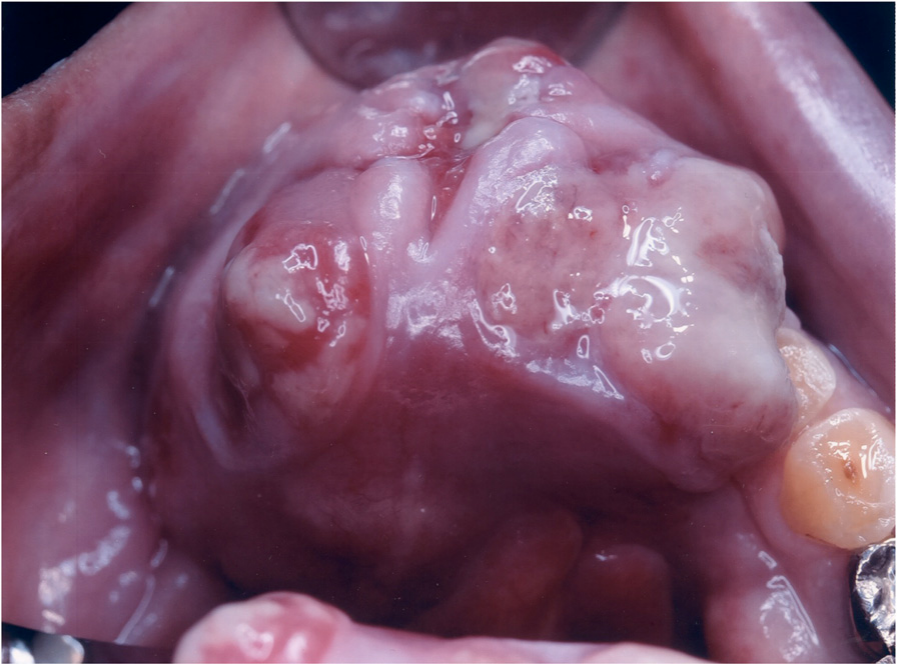
Intraoral tumor of the patient. The tumor mass measuring 2.6 × 2.1 cm in size rapidly enlarged in the medial mandibular gingiva

A 64-year-old man with gross swelling in the anterior mandibular gingiva for 1 month after lower incisal teeth extraction was referred to us for a closer evaluation. Oral examination revealed a firm mass measuring 2.6 × 2.1 cm in size arising from the extraction site (Fig. 1). The surface of the mass lesion showed erosion at the site equivalent to the wounds after tooth extraction. There was no hemorrhage or paralysis in the mandibular region, and the condition was painless.

For the past medical history, the patient underwent low anterior resection of a rectal malignancy diagnosed as moderately differentiated adenocarcinoma 7 years previously. There was no evidence of regional lymph node and distant metastases. Three years after the primary surgery, the patient showed bilateral lung metastases. Thus, the middle lobe of the right lung and the lower lobe of the left lung were partly resected. Four years later, multiple lung metastases and metastases to the vertebral bodies of the VI–VIII vertebrae were observed. The patient received 60 Gy of irradiation to control the pain from the lesion involving the vertebra.

**Fig. 2:**
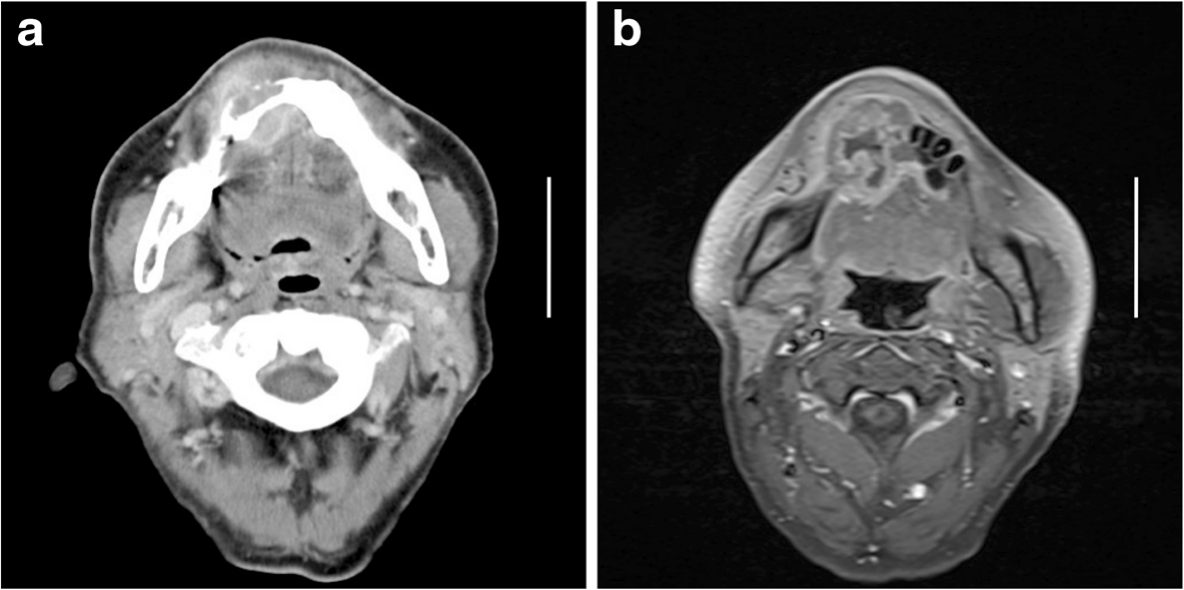
CT and MRI images of the mandibular region (scale bar, 5 cm). **a** A CT image showing a solid mass in the medial mandibular gingiva with bone destruction. **b** An MRI image showing a heterogeneous signal on a T1-weighted image after gadolinium administration

Computed tomography (CT) scan of the head and neck showed a solid mass and bony resorption in the anterior alveolar ridge of the mandible (Fig. 2a). There was no lymphadenopathy. Magnetic resonance imaging (MRI) revealed a heterogeneous signal on a T1-weighted image after gadolinium administration and an intense signal on a diffusion-weighted image (Fig. 2b).

**Fig. 3:**
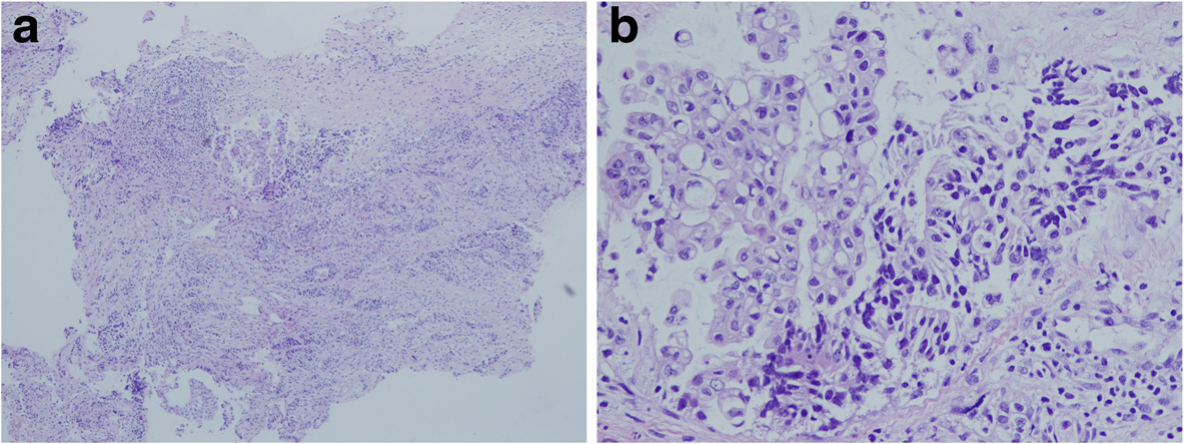
Microscopic appearance of the metastatic oral tumor. The biopsy specimen obtained at the first visit to our department shows moderately differentiated adenocarcinoma with a papillary tubular pattern comprising columnar to polygonal epithelial cells (hematoxylin and eosin stain, original magnifications ×40 (**a**) and ×200 (**b**))

**Fig. 4:**
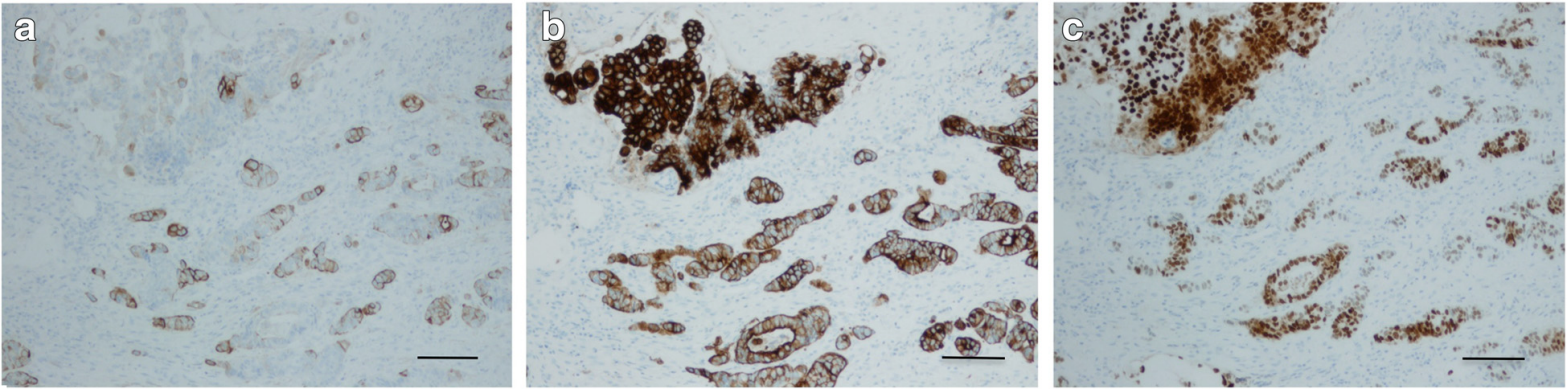
Immunohistochemical staining of the metastatic oral tumor showing tumor cells negative for CK7 (**a**), positive for CK20 (**b**), and positive for CDX2 (**c**) (original magnifications for all images, ×200; scale bar, 100 μm)

For the laboratory examination results, the serum carcinoembryonic antigen and cancer antigen 19-9 levels were 947.6 ng/ml and 1059.2 U/ml, respectively. Pathological examination by incisional biopsy of the gingival mass lesion revealed a moderately differentiated adenocarcinoma. The tumor was composed of proliferating columnar to polygonal epithelial cells with moderate atypia that showed a papillary tubular pattern (Fig. 3a, b). Immunohistological analysis demonstrated that the adenocarcinoma cells were positive for cytokeratin 20 (CK20) and negative for cytokeratin 7 (CK7) and positive for caudal type homeobox transcription factor 2 (CDX2) (Fig. 4a–c). The biopsy findings from the mandibular mass lesion were consistent with those from the resected rectal carcinoma 7 years previously.

The tumor had grown gradually and an ulcer was formed on the surface, making mastication difficult. The patient received palliative radiotherapy for the mandibular mass lesion. However, once the tumor showed a tendency to decrease in size, pneumonia developed and the patient’s cachexia deteriorated. Thus, the radiotherapy was discontinued, but the patient unfortunately died 2 months postradiotherapy.

### Discussion

Oral metastatic tumors are uncommon and comprise approximately 1 % of malignant oral neoplasms [1]. Most cases were previously diagnosed in reference to a primary neoplasm and were treated accordingly. Therefore, the primary site is usually assumed based on the past clinical history. However, 23 % of oral metastases were shown to be the first sign of the metastatic process [2]. In the present case, the origin of the metastatic lesion was clarified based on the past history of rectal adenocarcinoma resection and the subsequent lung metastases. The most common primary site in women is the breast followed by the female genital tract, kidney, and colon-rectum. In men, the most common primary site is the lung followed by the kidney, liver, and prostate [2].

In the oral site where metastatic tumors arise, most tumor lesions are observed in the jawbones, and only 16 % are found in the soft tissues such as the gingiva [3]. The metastatic rate in the posterior body of the mandible is higher than that in the symphyseal region [5]. The gingivae (55 %) followed by the tongue (30 %) are the most common soft tissue sites affected by metastatic malignancy [4]. In the present case, the metastatic lesion has been presumed to develop in the gingival tissue of the symphysis. This lesion was an exophytic growing mass, and the radiological appearance of the mandibular bone invasion was not demonstrated apparently. Likewise, some case reports have shown that the metastatic site was the gingiva for the primary lesion of colonic and rectal adenocarcinomas [6–8].

Adenocarcinoma of the colon and rectum commonly spreads to the regional lymph nodes, liver, and lungs, but less commonly to the peritoneum, bone, adrenal glands, brain, kidney, thyroid, pancreas, ovaries, and skin. Clausen and Poulsen proposed the following three criteria for metastatic oral tumor: *firstly*, the metastatic tumor is pathologically similar to the primary tumor; *secondly*, the oral tumor is considered to be a metastasis clinically and pathologically; and *thirdly*, the oral tumor is atypical compared with common oral primary tumors [9]. The most common primary tumor arising from the oral mucosa is squamous cell carcinoma. Therefore, there may be a need to differentiate metastatic adenocarcinoma from adenocarcinoma derived from the salivary gland, although the frequency of occurrence is very low. Three clinical courses may be considered to underlie the metastasis of colonic adenocarcinoma to the oral cavity as follows: (1) direct metastasis with identification of the primary tumor, (2) secondary metastasis derived from metastasis in another site after treatment of the primary tumor, and (3) occult metastasis without identification of the primary tumor. The present case follows the second clinical course in which the secondary metastasis in the jawbone was derived from multiple lung metastatic lesions. The primary rectal tumor has already been resected completely. The present case conformed to Clausen’s criteria.

Histopathologically, approximately 70 % of the metastatic lesion in the oral region is reportedly adenocarcinoma, followed with much less cases by clear cell carcinoma from the kidney and squamous cell carcinoma from the lungs [10]. Also in the present case, the hematoxylin and eosin-stained section showed features of adenocarcinoma arising from the rectum. Furthermore, the histological diagnosis was supported by the immunohistochemical analysis. The metastatic tumor cells demonstrated a CK20-positive and a CK7-negative phenotype. In the evaluation of gastrointestinal tract carcinomas, the great majority of well-differentiated or moderately differentiated large intestinal adenocarcinomas reportedly showed a CK7-negative/CK20-positive phenotype. Adenocarcinomas of the upper gastrointestinal tract were also reported to be positive for both CK7 and CK20 in 78 % of cases [11]. Thus, an assessment of the CK7 and CK20 phenotype may help in determining the primary site of a metastatic tumor [11]. In addition to the CK7 and CK20 immunoprofiles, the tumor cells showed CDX2 immunoreactivity. CDX2 is a sensitive and specific maker for adenocarcinoma with a colorectal origin [12]. CDX2 expression has also been shown in a subset of adenocarcinomas arising from the colon, rectum, stomach, esophagus, and ovary [13]. Accordingly, CK7, CK20, and CDX2 may be useful for the diagnosis of metastatic tumors of gastrointestinal origin.

In the case of oral metastatic tumors, they usually have a poor prognosis because often they have already spread to other sites. Therefore, the appropriate palliative treatment has to be carefully selected. If the tumor is widely disseminated, palliative radiotherapy is recommended [10]. However, when possible, surgical resection may be recommended mostly for metastatic lesions in the soft oral tissue [10]. In the present case, as the oral metastatic lesion was rapidly growing with accompanying multiple lung metastases, resection was not possible under general anesthesia. Accordingly, we performed palliative radiotherapy for the mandibular mass. Alternatively, chemotherapy with the FOLFIRI regimen (5-FU, leucovorin, irinotecan) is usually performed for advanced metastatic colorectal carcinoma [6]. Although the IRIS regimen (irinotecan, S-1) plus bevacizumab (monoclonal antibody targeted against vascular endothelial growth factor) was planned when the lung metastasis occurred to play the role of another second-line chemotherapeutic regimen, the patient showed no response to the chemotherapy.

Patients with distant metastasis of colorectal adenocarcinoma usually have a poor prognosis. In particular, if a secondary metastatic oral tumor is identified, the disease state may be commonly progressive and the prognosis is extremely poor. In cases involving metastasis to the oral cavity, the mean survival period from the time of mass appearance to the time of death of a patient has been reported to be about 7 months [2]. Regarding metastasis to the gingiva, some cases have been documented showing that the duration of survival from the indication of gingival mass development was only within 1 year [6, 14, 15]. The present case also showed a short survival period of 4 months.

## Conclusions

We present the case of a patient with metastatic rectal adenocarcinoma to the mandibular gingiva. The metastatic oral tumor has developed as a secondary metastasis 7 years following the resection of the primary rectal tumor. This case indicates that careful clinical and pathological evaluations are important in making a definitive diagnosis particularly in patients with a past history of a malignant lesion. When metastasis to the oral region is confirmed, the patient is often already in the advanced stage. In such a patient, therapeutic management that includes palliative treatment must be considered.

## Abbreviations

CDX2, caudal type homeobox transcription factor 2; CK20, cytokeratin 20; CK7, cytokeratin 7; CT, computed tomography; MRI, magnetic resonance imaging

## References

[1] Meyer I, Shklar G (1965). Malignant tumors metastatic to mouth and jaw. Oral Surg Oral Med Oral Pathol.

[2] Hirshberg A, Shnaiderman-Shapiro A, Kaplan I, Berger R (2008). Metastatic tumours to the oral cavity—pathogenesis and analysis of 673 cases. Oral Oncol.

[3] Hirshberg A, Buchner A (1995). Metastatic tumours to the oral region, an overview. Oral Oncol Eur J Cancer.

[4] Thomaz LA, Duarte MT, de Camargo de Moraes P, de Araujo VC, Soares AB (2011). Metastatic adenocarcinoma of the colon: early manifestation in gingival tissue. Head Neck Pathol.

[5] Zohar Y, Ben-Tovim R, Gal R (1985). Metastatic carcinoma of oral soft tissue. Head Neck Surg.

[6] Iida T, Sasaki T, Akita H, Sasaki M, Shiba H, Tanaga K (2009). Metastatic gingival tumor from rectal cancer diagnosed with CDX2. Clin J Gastroenterol.

[7] Favia G, Maiorano E, Muzio LL (2010). Gingival metastasis from colonic adenocarcinoma. Clin Gastroenterol Hepatol.

[8] Soares AB, Thomaz LA, Duarte MT, de Camargo de Moraes P, de Araújo VC (2011). Metastatic adenocarcinoma of the colon: early manifestation in gingiva tissue. Head Neck Pathol.

[9] Clausen F, Poulsen H (1963). Metastatic carcinoma to the jaws. Acta Path Microbiol Scand.

[10] van der Waal RIF, Buter J, van der Waal I (2003). Oral metastases: report of 24 cases. Br J Oral Maxillofac Surg.

[11] Kende AI, Carr NJ, Sobin LH (2003). Expression of cytokeratins 7 and 20 in carcinomas of the gastrointestinal tract. Histopathology.

[12] Kaimaktchiev V, Terracciano L, Tornillo L, Spichtin H, Stoios D, Bundi M (2004). The homeobox intestinal differentiation factor CDX2 is selectively expressed in gastrointestinal adenocarcinomas. Mod Pathol.

[13] Dennis JL, Hvidsten TR, Wit EC, Komorowski J, Bell AK, Downie I (2005). Markers of adenocarcinoma characteristic of the site of origin: development of a diagnostic algorithm. Clin Cancer Res.

[14] Murugaraj V, Bischoff P, Fasanya S, Hameed S, Suman A (2013). Metastatic colorectal adenocarcinoma of the jaw—a case report. Oral Surg.

[15] Álvarez CA, Rodríguez BI, Irazu SP, Sánchez-Gracián CD (2006). Colon adenocarcinoma with metastasis to gingiva. Med Oral Pathol Oral Cir Bucal.

